# Elevated RBP4 plasma levels were associated with diabetic retinopathy in type 2 diabetes

**DOI:** 10.1042/BSR20181100

**Published:** 2018-09-12

**Authors:** Jia-Ying Li, Xian-Xian Chen, Xiao-Hua Lu, Chuang-Biao Zhang, Qi-Ping Shi, Lie Feng

**Affiliations:** Department of Endocrinology, The First Affiliated Hospital of Jinan University, Guangzhou 510632, China

**Keywords:** Chinese, diabetes mellitus, diabetic retinopathy, retinol-binding protein 4

## Abstract

The retinol-binding protein 4 (RBP4) has been postulated to play a role in glucose homeostasis, insulin resistance, and diabetes mellitus in human and animal studies. The aim of the present study was to evaluate the role of RBP4 in Chinese patients with type 2 diabetes mellitus with and without diabetic retinopathy (DR). Plasma RBP4 concentrations were tested in 287 patients with type 2 diabetes. At baseline, demographic and clinical information including presence of DR and vision-threatening DR (VTDR) was collected. The relationship between RBP4 and DR (VTDR) was investigated using logistic regression. Patients with DR or VTDR had significantly higher plasma levels of RBP4 on admission (*P*<0.0001). Receiver operating characteristics (ROCs) to predict DR and VDTR demonstrated areas under the curve for RBP4 of 0.79 (95% confidence interval (CI): 0.73–0.85) and 0.90 (95% CI: 0.85–0.94), respectively, which were superior to other factors. For each 1 μg/ml increase in plasma level of RBP4, the unadjusted and adjusted risk of DR would be increased by 8% (with the odds ratio (OR) of 1.08 (95% CI: 1.05–1.13), *P*<0.001) and 5% (1.05 (1.02–1.11), *P*=0.001), respectively. It was 12% (with the OR of 1.12 (95% CI: 1.07–1.18), *P*<0.001) and 9% (1.09 (1.05–1.15), *P*<0.001) for VTDR. The present study shows that elevated plasma levels of RBP4 were associated with DR and VDTR in Chinese patients with type 2 diabetes, suggesting a possible role of RBP4 in the pathogenesis of DR complications. Lowering RBP4 could be a new strategy for treating type 2 diabetes with DR.

## Introduction

The world’s largest diabetes epidemic is in China [1]. The prevalence of diabetes in China was continuing to increase, which was reported to be 0.67% in 1980 and 10.9% in the latest published nationwide estimate in 2013 [2]. The major complication of diabetes is diabetic retinopathy (DR), a leading cause of visual impairment and blindness worldwide [3]. In a population-based cross-sectional study, Wang et al. [4] found that the overall prevalence of DR was 43.1% in diabetes mellitus, and the prevalence of proliferative DR (PDR), macular edema, and vision-threatening retinopathy was 1.6, 5.2, and 6.3%, respectively. Longer diabetes duration and poorer glycemic and blood pressure control play a key role in DR, but the maintenance of normoglycemia does not always completely prevent its development [3,5]. Therefore, other factors associated with the diabetic state are postulated to play a role in DR.

Retinol-binding protein 4 (RBP4) protein belongs to the lipocalin family and is the specific carrier for retinol (vitamin A) in the blood [6]. It was reported that the level of circulating RBP4 is often elevated in obese mice and humans and that, under these circumstances, the protein induces insulin resistance [7]. Previous studies had suggested that circulating RBP4 levels were associated with insulin resistance [8], metabolic syndrome [9], impaired glucose tolerance [10], and type 2 diabetes [11].

Intestinally, Huang et al. [12] reported that RBP4 could impair pancreatic β-cell function, which may lead to the onset and the development of type 2 diabetes. Another study suggested that RBP4 marker may serve as a tool to follow-up clinical monitoring of the development and progression of diabetic nephropathy (DN) [13]. However, the role of RBP4 in DR was not fully understood. Only a small study including 92 patients with type 2 diabetes described that RBP4 may be a novel biomarker for its diagnosis and treatment in diabetic patients [14]. Therefore, we speculated that plasma RBP4 might be associated with DR in Chinese patients with type 2 diabetes. The aim of the present study was to investigate the relationship between plasma RBP4 levels and DR, and to determine its possible role in type 2 diabetes.

## Methods

From January 2016 to December 2017, all patients with type 2 diabetes from Department of Endocrinology of The First Affiliated Hospital of Jinan University (Guangzhou, China) were screened for the study. Type 2 diabetes was defined according to the criteria of the American Diabetes Association (a glycated hemoglobin (HbA1c) level ≥ 6.5% and/or a fasting plasma glucose (FPG) ≥ 7.0 mmol/l) [15]. Patients with the following conditions were excluded: (i) malignant tumor; (ii) metabolic syndrome (not including type 2 diabetes); (iii) liver and renal insufficiency (creatinine (Cr) > 1.5 mg/dl); (iv) cardio-cerebral vascular disease and neurological disorders; and (v) ongoing infection or autoimmune diseases. Patients, who had no light perception (severe visual impairment) or had a severe infection in one or both eyes, were also excluded.

In addition, 150 normal people without type 2 diabetes from the Physical Examination Center were designated as the control group. Those people matched with the patients for sex, age, and body mass index (BMI). The study followed the principles of the Declaration of Helsinki and was approved by the Institute Ethics Committee of The First Affiliated Hospital of Jinan University. All participants were informed of the study protocol and their written informed consents were obtained.

The following demographic and clinical data including age, sex, race, BMI, systolic and diastolic blood pressure, diabetes duration, presence and severity of DR, diabetic macular edema (DME) status, hypertension, hyperlipoproteinemia, smoking habits, alcohol abuse, and treatments (insulin, hypoglycemic drugs, lipid lowering, and blood pressure lowering) were recorded at admission.

The Canon CR6-45NM ophthalmic digital imaging system and a Canon EOS 10D digital camera were used to take two digital images per eye through a non-pharmacologically dilated pupil (X.-X.C. and X.-H.L.). DR was diagnosed according to the presence of one or more retinal microaneurysms or retinal blot hemorrhages by the Early Treatment Diabetic Retinopathy Study (ETDRS) grading standards [16]. The DR severity score was assigned to each eye according to the modified Airlie House classification system [17]. If both eyes were rated at different stages, then the grade of the worst eye was used. DR was divided into two groups as non-PDR (NPDR; levels 20–59) and PDR (levels ≥ 60). DME was defined as present or absent and classified as with or without clinically significant DME. Vision-threatening DR (VTDR) was defined as the presence of PDR and/or DME.

The fasting blood samples were collected on the first day of admission before any treatment had been implemented. After centrifugation, the plasma samples were immediately stored at −80°C before assay. Plasma RBP4 levels were batch analyzed using a commercially available ELISA assay (R&D Systems, Minneapolis, MN, U.S.A.). The detection range for RBP4 was 1.6–100 ng/ml. Inter-assay and intra-assay coefficients of variation were 4.5–9.0% and 4.0–7.0%, respectively. In the present study, a 1:100 dilution was used, due to the high levels of RBP4 (μg/ml) in the plasma sample. The median plasma level of RBP4 in the 150 normal individuals was 15.5 μg/ml and the 97.5th percentile was 28.5 μg/ml. Blood levels of high-sensitivity C-reactive protein (Hs-CRP), HbA1c, insulin, FPG, and Cr were tested using routine laboratory methods by ROCHE COBASC311 (Roche, Basel, Switzerland). The urinary albumin excretion (UAE) rate was measured by enzyme immunoassay using immunoturbidimetry with a 24-h urine collection (Eiken, Tokyo, Japan). The homeostasis model assessment of insulin resistance (HOMA-IR) index was calculated from the fasting blood glucose and fasting plasma insulin concentrations by the formula: HOMA-IR = fasting plasma insulin (μU/ml) × fasting blood glucose (mmol/l)/22.5 [18]. Estimated glomerular filtration rate (eGFR) was calculated by an equation for Chinese: eGFR (ml/min/1.73 m^2^) = 175 × Cr-1.234 age-0.179 sex (male = 1, female = 0.19).

### Statistical analysis

Categorical variables were expressed as percentages and continuous variables were expressed as medians (interquartile ranges, IQRs). Differences between the two groups were compared by Chi-Square or Mann–Whitney U-test as appropriate. Correlations amongst continuous variables were tested by the Spearman’s rank correlation coefficient.

To investigate whether RBP4 allows prediction of both DR and VTDR in diabetes, different statistical methods were used. First, the relation of RBP4 with the two points was investigated with the use of logistic regression models. We used crude models and multivariate models adjusted for all significant predictors and report odds ratios (ORs). For multivariate analysis, we included confounders, known risk factors, and other predictors as assessed in univariate analysis. For a more detailed exploration of the RBP4 and DR (VTDR), we also used multivariate analysis models to estimate adjusted OR and 95% confidence intervals (CIs) of DR (VTDR) for RBP4 quartiles (with quartiles 1–3 as reference). Second, receiver operating characteristic (ROC) curves were used to test the overall prediction accuracy of RBP4 and other markers, and results were reported as area under the curve (AUC). In addition, the predicted accuracy of combined model I (RBP4/HOMA-IR/diabetes duration) and combined model II (RBP4/HOMA-IR/diabetes duration/Hs-CRP/HbA1c) were calculated. All statistical analysis were performed with SPSS for Windows, version 22.0 (SPSS Inc., Chicago, IL, U.S.A.) and the ROCR package (version 1.0-2). Statistical significance was defined as *P*<0.05.

## Results

### Patient characteristics and clinical variables

In the present study, 287 patients with type 2 diabetes were included. The median age was 62 (IQR: 55–71) years and 155 (54.0%) were men. The diabetes duration of those patients was 12 (IQR: 6–15) years. One hundred and twenty-one (42.2%) out of 287 were taking insulin and 159 were taking oral hypoglycemic agents. The plasma levels of insulin in patients, who were taking insulin, were not different when compared with those who were not taking insulin (9.68 (IQR: 6.45–12.15) compared with 9.49 (6.38–12.04); *P*=0.33). Amongst those patients, 83 were defined as DR (28.9%; 95% CI: 23.7–34.2%). As shown in the Table 1, patients with DR had higher levels of HbA1c, Hs-CRP, fasting glucose, HOMA-IR, and diabetes duration than those without DR (*P*<0.05). In addition, intensive glucose-lowering therapy was more common in patients with DR than in those without DR (*P*<0.05). Basal characteristic of diabetes patients with DR or without DR were listed in the Table 1.

**Table 1:** Basal characteristic of diabetes patients with DR or without DR

	Diabetes	Retinopathy status		
Characteristics	*n*=287	Yes (*n*=83)	No (*n*=204)	*P*^1^
Age at admission (IQR, years)	62 (55–71)	61 (55–72)	62 (55–70)	0.76
Male, *n* (%)	155 (54.0)	48 (57.8)	107 (52.5)	0.41
Race-Han, *n* (%)	257 (89.5)	74 (89.2)	183 (89.7)	0.89
BMI (IQR, kg/m^2^)	26.8 (24.3–28.8)	27.5 (25.9–9.4)	26.5 (24.3–27.4)	0.066
Diabetes duration (IQR, years)	12 (6–15)	14 (8–16)	11 (5–13)	0.001
Systolic blood pressure (IQR, mmHg)	135 (125–140)	139 (128–144)	132 (122–135)	0.093
Smoking status, *n* (%)	76 (26.5)	24 (28.9)	52 (25.5)	0.55
Current alcohol intake, *n* (%)	69 (24.0)	22 (26.5)	47 (23.0)	0.53
Intensive glucose treatment^2^ (%)	136 (47.4)	51 (61.4)	85 (41.7)	0.002
Use of lipid-lowering medication (%)	101 (35.2)	31 (37.3)	70 (34.3)	0.63
HOMA-IR (IQR)	4.65 (2.21–5.69)	5.34 (2.54–6.44)	3.88 (1.69–5.15)	<0.001
Laboratory findings (IQR)				
HbA1c (%)	7.2 (6.2–8.6)	8.5 (7.0–9.5)	6.9 (5.9–8.1)	<0.001
Hs-CRP (mg/dl)	0.79 (0.30–1.28)	0.90 (0.40–1.66)	0.69 (0.25–0.98)	0.001
Fasting insulin (uU/ml)	9.55 (6.40–12.11)	10.46 (7.19–13.82)	8.68 (5.76–9.92)	0.009
FBG (mmol/l)	6.15 (5.10–7.88)	6.83 (5.57–8.39)	5.83 (4.86–7.03)	0.012
eGFR (ml/min/1.73 m^2^)	130 (102–155)	137 (104–159)	127 (100–150)	0.21
UAE (mg/day)	10.1 (6.2–20.2)	10.7 (6.4–20.6)	9.8 (6.0–19.9)	0.103
RBP4 (μg/ml)	28.6 (18.1–38.0)	39.7 (29.8–47.7)	25.0 (15.5–32.6)	<0.001
Any DR, *n* (%)	83 (28.9)	—	—	—
PDR	25 (8.7)			
DME	21 (7.3)			
VTDR	39 (13.6)			

Results are expressed as percentages or as medians (IQR). Abbreviation: FBG, fasting blood glucose.

1 *P*-values were compared by Mann–Whitney U-test or Chi-Square test as appropriate.

2 Sulphonyl urea or insulin or, if more than 120% of ideal body weight, metformin.

The data suggested that plasma levels of RBP4 in patients with diabetes were significantly higher than in normal controls (28.6 (IQR: 18.1–38.0) μg/ml compared with 15.5 (IQR: 11.0–22.8) μg/ml; *P*<0.001). In those patients with diabetes, plasma levels of RBP4 were positively correlated with HbA1c (r = 0.347, *P*<0.0001), BMI (r = 0.163, *P*=0.001), Hs-CRP (r = 0.302, *P*<0.001), and duration of illness (r = 0.179, *P*<0.001). Furthermore, RBP4 was significantly correlated with the HOMA-IR (r = 0.628, *P*<0.0001).

### RBP4 and DR

**Figure 1:**
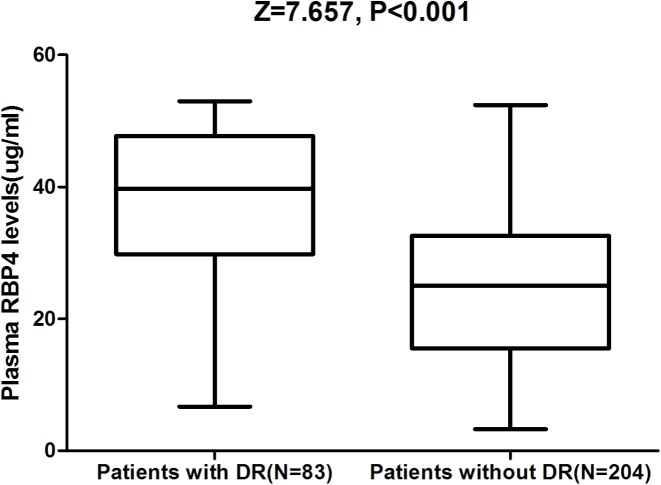
The plasma levels of RBP4 between diabetes with DR and without DR The horizontal lines indicate median levels and IQR. *P*-values refer to Mann–Whitney U-tests for differences between groups.

Plasma levels of RBP4 were significantly higher in patients with DR than those without DR (39.7 (IQR: 29.8–47.7) μg/ml compared with 25.0 (IQR: 15.5–32.6) μg/ml; *P*<0.001; Figure 1). In univariate and multivariate logistic regression analysis, we calculated the OR of RBP4 compared with other risk factors as presented in Table 2. For each 1 μg/ml increase in plasma level of RBP4, the unadjusted and adjusted risk of DR would be increased by 8% (with the OR of 1.08 (95% CI: 1.05–1.13), *P*<0.001) and 5% (1.05 (1.02–1.11), *P*=0.001), respectively (Table 2). Furthermore, there was an increased risk of DR associated with RBP4 concentrations ≥38.0 μg/ml (>third quartile; OR = 3.44, 95% CI: 2.39–5.76; *P*<0.001) after adjusting for possible confounders. In addition, unlike other factors, HbA1c, Hs-CRP, and HOMA-IR were also identified as DR indicators in multivariate analysis (Table 2).

**Table 2:** Logistic regression model for RBP4 and other predictors using DR and VTDR as the dependent variables

Parameter	Univariate analysis			Multivariate analysis		
	OR	95% CI^1^	*P*	OR	95% CI^1^	*P*
Predictor: DR						
RBP4	1.08	1.05–1.13	<0.001	1.05	1.02–1.11	0.001
HbA1c	1.26	1.05–1.59	0.001	1.15	1.04–1.33	0.015
Hs-CRP	1.85	1.24–2.58	0.003	1.52	1.09–2.28	0.032
Diabetes duration	1.08	1.02–1.16	0.040	1.04	0.97–1.12	0.28
BMI	1.13	0.99–1.27	0.066	—		
HOMA-IR	1.22	1.15–1.29	<0.001	1.15	1.07–1.22	<0.001
FBG	1.19	1.04–1.40	0.018	1.08	0.99–1.20	0.19
Intensive glucose treatment^2^	2.15	1.58–3.06	0.002	1.59	1.18–2.44	0.030
Use of lipid-lowering medication	1.35	0.93–2.08	0.63	—		
Predictor: VTDR						
RBP4	1.12	1.07–1.18	<0.001	1.09	1.05–1.15	<0.001
HbA1c	1.44	1.23–1.78	<0.001	1.21	1.10–1.35	0.002
Hs-CRP	2.01	1.40–2.76	0.003	1.36	1.10–1.59	0.012
Diabetes duration	1.29	1.09–1.50	0.012	1.13	1.02–1.31	0.021
BMI	1.27	0.94–1.89	0.18	—		
HOMA-IR	1.53	1.28–1.85	<0.001	1.29	1.16–1.55	<0.001
FBG	1.30	1.13–1.65	0.012	1.15	1.01–1.50	0.27
Intensive glucose treatment^2^	1.85	1.29–2.85	0.011	1.59	1.04–2.25	0.13
Use of lipid-lowering medication	1.66	0.80–3.35	0.63	-		

1 Note that the OR corresponds to a unit increase in the explanatory variable.

2 Sulphonyl urea or insulin or, if more than 120% of ideal body weight, metformin.

Based on the ROC curve, the optimal cut-off value of plasma RBP4 as an indicator for DR diagnosis was projected to be 36.4 μg/ml, which yielded a sensitivity of 61.5% and a specificity of 83.3%, with an AUC of 0.79 (95% CI: 0.73–0.85; *P*<0.001). With this AUC, RBP4 presented a greater discriminatory ability to diagnose DR compared with HbA1c (AUC 0.71; 95% CI: 0.63–0.78; *P*<0.001), Hs-CRP (AUC 0.65; 95% CI: 0.59–0.72; *P*<0.001), diabetes duration (AUC 0.62; 95% CI: 0.55–0.69; *P*<0.001), and HOMA-IR (AUC 0.73; 95% CI: 0.68–0.80; *P*=0.005). Interestingly, the combined model I (RBP4/HOMA-IR/diabetes duration) improved the ability of every single marker to diagnose DR (AUC of the combined model 0.84; 95% CI: 0.78–0.90; *P*<0.01). Furthermore, the combined model II (RBP4/HOMA-IR/diabetes duration/Hs-CRP/HbA1c) improved the ability of every single marker and model I to diagnose DR (AUC of the combined model 0.87; 95% CI: 0.80–0.92; *P*<0.05).

### RBP4 and VTDR

**Figure 2:**
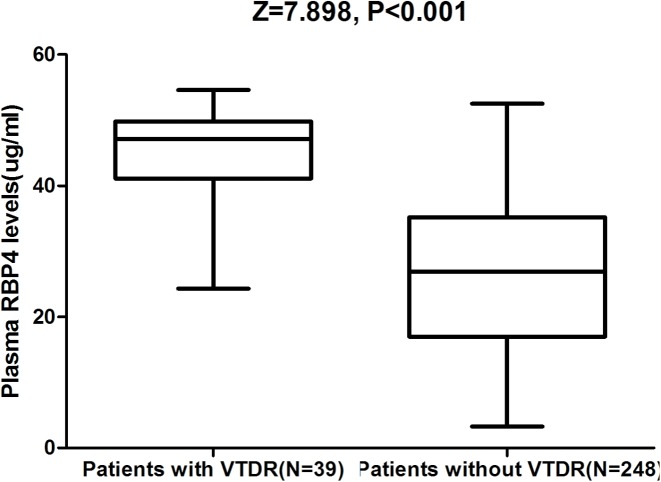
The plasma levels of RBP4 between diabetes with VTDR and without VTDR The horizontal lines indicate median levels and IQR. *P*-values refer to Mann–Whitney U-tests for differences between groups.

Thirty-nine patients were defined as VTDR; thus, the rate was 13.6% (95% CI: 9.6–17.6%). Plasma RBP4 levels were significantly higher in patients with VTDR than those without VTDR (47.1 (IQR: 41.1–49.8) μg/ml compared with 26.9 (IQR: 17.2–35.2) μg/ml; *P*<0.001; Figure 2). In univariate and multivariate logistic regression analyses, we calculated the OR of RBP4 compared with other risk factors as presented in Table 2. For each 1 μg/ml increase in plasma level of RBP4, the unadjusted and adjusted risks of VTDR would be increased by 12% (with the OR of 1.12 (95% CI: 1.07–1.18), *P*<0.001) and 9% (1.09 (1.05–1.15), *P*<0.001), respectively (Table 2). Furthermore, there was an increased risk of VTDR associated with RBP4 concentrations ≥ 38.0 μg/ml (>third quartile; OR = 3.89, 95% CI: 2.55–6.18; *P*<0.001) after adjusting for possible confounders. In addition, unlike other factors, HbA1c, Hs-CRP, HOMA-IR, and diabetes duration were also identified as VTDR indictors in multivariate analysis (Table 2).

Based on the ROC curve, the optimal cut-off value of plasma RBP4 as an indicator for VTDR diagnosis was projected to be 38.4 ug/ml, which yielded a sensitivity of 87.2% and a specificity of 82.6%, with an area AUC of 0.90 (95% CI: 0.85–0.94; *P*<0.001). With this AUC, RBP4 presented a greater discriminatory ability to diagnose VTDR compared with HbA1c (AUC 0.76; 95% CI: 0.70–0.82; *P*<0.001), Hs-CRP (AUC 0.71; 95% CI: 0.65–0.78; *P*<0.001), diabetes duration (AUC 0.77; 95% CI: 0.71–0.83; *P*<0.001), and HOMA-IR (AUC 0.80; 95% CI: 0.74–0.86; *P*<0.001). Interestingly, the combined model (RBP4/HOMA-IR/diabetes duration) improved the ability of every single marker to diagnose VTDR (AUC of the combined model 0.93; 95% CI: 0.86–0.96; *P*<0.01). Furthermore, the combined model II (RBP4/HOMA-IR/diabetes duration/Hs-CRP/HbA1c) did not improved the ability of model I to diagnose VTDR (AUC of the combined model 0.94; 95% CI: 0.86–0.96; *P*>0.05).

## Discussion

Plasma levels of RBP4, a protein secreted by adipocytes, are increased in insulin-resistant states and are associated with cardiovascular risk factors in subjects with varied clinical presentations [19]. RBP4 also had been suggested to be associated with variables related to insulin resistance and diabetic complications [20]. Our main findings were the following: (i) plasma levels of RBP4 were higher in patients with type 2 diabetes compared with normal controls; (ii) RBP4 plasma levels were associated with DR (VTDR) independently of a broad range of routine risk factors; (iii) patients in the top quartile of RBP4 had a 3.44 (3.89)-fold greater risk of developing DR (VTDR) compared with those in the other three quartiles.

A previous study reported that the occurrence rate of DR and VTDR in patients with type 2 diabetes was 28.1 and 13.2%, respectively [3]. Consistent with this, in the present study, we found that 28.9 and 13.6% of the diabetes suffered from DR and VTDR. In eastern China communities, Pan et al. [21] reported that the overall prevalence of any DR and VTDR was 18.0 and 4.4%, respectively. Furthermore, the prevalence of DR, NPDR, and PDR in adult Chinese American individuals was reported as 35.8, 1.7, and 2.4%, respectively [22]. The general discrepancy between DR prevalence rates might be caused by the differences in subject samples, diagnostic assessment methods, and time of assessment.

Lots of potential biomarkers, such as mannose-binding lectin [23], brain-derived neurotrophic factor [24], fatty acid binding protein 4 [25], lipoprotein (a) [26], and miRNA [27–28] had been suggested to be associated with DR. Herein, we proposed that RBP4 also was a potential biomarker for DR. Consistent with our findings, another study showed that RBP4 may be involved in the process of DR [14]. Furthermore, RBP4 also had been suggested as a useful marker of overall metabolic control in Chinese patients with type 2 diabetes mellitus [29]. In addition, Murata et al. [30] indicated an increase in serum RBP4 levels in the type 2 diabetic subjects, particularly complicated with advanced renal impairment.

Interestingly, elevated plasma RBP4 concentration might be a biomarker of nephropathy and cardiovascular disease in type 2 diabetic subjects [31]. Another study showed that elevated RBP4 levels in type 2 diabetic patients may be the result of moderate renal insufficiency [32], while Raila et al. [33] suggested that plasma RBP4 levels in type 2 diabetic patients are affected by incipient nephropathy. However, we did not find a correlation between RBP4 and eGFR. This may in part be caused by the fact that patients with renal insufficiency were excluded from our study to eliminate any effect of abnormal renal function on RBP4 metabolism. Further studies should be carried out to examine the association between RBP4 and kidney disease.

Diabetes duration, glycemic control [3], and inflammation [34] were associated with DR. Similarly, we also found that diabetes duration, HbA1c, and Hs-CRP, were associated with DR. RBP4 might play a role in the DR through those factors. However, after adjusting those factors in the multivariate logistic regression analysis, RBP4 still associated with DR, suggesting that other possible mechanisms need to be considered. First, plasma RBP4 levels were associated with an adverse profile of oxidative stress and inflammatory markers [35]. Interestingly, oxidative stress had been suggested to play a role in the pathogenesis of DR [36]. Second, a previous study suggested that baseline RBP4 predicted insulin resistance (OR  =  1.44, *P*=0.015) in the 10-year follow-up phase in Beijing Child and Adolescent Metabolic Syndrome study [37]. In fact, insulin resistance was an independent specific marker of proliferative retinopathy [38]. Lastly, Farjo et al. [39] revealed that RBP4 elevation can directly contribute to endothelial inflammation, and therefore may play a causative role in the development or progression of vascular inflammation during cardiovascular disease and microvascular complications of diabetes.

Some limitations should be considered when interpreting the result. First, without serial measurement of circulating RBP4, when and how long RBP4 affected our participants were not tested. In the present study, 121 patients were taking insulin and their serum insulin levels were measured for the calculation of HOMA-IR. Their insulin levels might be not properly accurate because they were taking insulin. However, the fasting blood samples were collected before insulin treatment. In addition, plasma levels of insulin in patients taking insulin were not different when compared with those patients not taking insulin. Second, the cross-sectional design does not allow for cause or effect relationships. Third, some patients with severe visual impairment (such as advanced DR) had been excluded. Fourth, genetic variants in the RBP4 gene may be associated with circulating RBP4 concentration and phenotypes related to glucose metabolism [40]. However, we did not test the RBP4 genetic variants. Lastly, the use of ELISA kits, with limited dynamic range, could result in conflicting results. Commercial ELISA kits do not differentiate between holo- and apo-RBP4 and evaluate the whole RBP4 concentration.

## Conclusion

The present study shows that elevated plasma levels of RBP4 were associated with DR and VDTR in Chinese patients with type 2 diabetes, suggesting a possible role of RBP4 in the pathogenesis of DR complications. Further studies are required to validate our findings and elucidate the causal relationship of RBP4 in the pathology of the DR. Lowering RBP4 could be a new strategy for treating type 2 diabetes with DR.

## Abbreviations

AUC: area under the curve
BMI: body mass index
CI: confidence interval
Cr: creatinine
DME: diabetic macular edema
DR: diabetic retinopathy
eGFR: estimated glomerular filtration rate
FPG: fasting plasma glucose
HOMA-IR: homeostasis model assessment of insulin resistance
Hs-CRP: high-sensitivity C-reactive protein
IQR: interquartile range
NPDR: non-proliferative DR
OR: odds ratio
PDR: proliferative DR
ROC: receiver operating characteristic
RBP4: retinol-binding protein 4
VTDR: vision-threatening DR
